# The RND Efflux Pump Gene Expression in the Biofilm Formation of *Acinetobacter baumannii*

**DOI:** 10.3390/antibiotics12020419

**Published:** 2023-02-20

**Authors:** Ola A. Abd El-Rahman, Fatma Rasslan, Safaa S. Hassan, Hossam M. Ashour, Reham Wasfi

**Affiliations:** 1 Department of Microbiology and Immunology, Faculty of Pharmacy (Girls), Al-Azhar University, Cairo 11751, Egypt; 2 Department of Clinical Pathology, National Cancer Institute, Cairo University, Cairo 11796, Egypt; 3 Department of Integrative Biology, College of Arts and Sciences, University of South Florida, St. Petersburg, FL 33701, USA; 4 Department of Microbiology and Immunology, Faculty of Pharmacy, October University for Modern Sciences and Arts (MSA), Giza 12451, Egypt

**Keywords:** *Acinetobacter baumannii*, biofilm, RND efflux pump, AdeABC, AdeFGH, AdeIJK

## Abstract

Multidrug resistant (MDR) *Acinetobacter baumannii* is a critical opportunistic pathogen in healthcare-associated infections (HAI). This is attributed to several factors, including its ability to develop biofilms that can enhance antimicrobial resistance (AMR) in addition to creating an environment for horizontal transfer of antibiotic resistance genes. The role of the efflux pump in biofilm formation is important for studies on alternative treatments for biofilms. One of the significant efflux pump families is the RND efflux pump family, which is common in Gram negative bacteria. The aim is to study the role of the RND efflux pump in biofilm formation by *A. baumannii*. The biofilm formation potential of thirty-four MDR *A. baumannii* isolates was evaluated by crystal violet assays. The effect of efflux pump inhibition and activation was studied using the efflux pump inhibitor carbonyl cyanide 3-chlorophenylhydrazone (CCCP) and the RND efflux pump substrate levofloxacin (at sub-MIC), respectively. The isolates were genotypically grouped by enterobacterial repetitive intergenic consensus (ERIC) typing and the expression of *adeABC*, *adeFGH*, and *adeIJK* efflux pump genes was measured by qPCR. Overall, 88.2% (30/34) of isolates were biofilm producers (the phenotype was variable including strong and weak producers). Efflux pump inhibition by CCCP reduced the biofilm formation significantly (*p* < 0.05) in 17.6% (6/34) of some isolates, whereas sub-MICs of the substrate levofloxacin increased biofilm formation in 20.5% (7/34) of other isolates. Overexpression of the three RND efflux pump genes was detected in five out of eleven selected isolates for qPCR with remarkable overexpression in the *adeJ* gene. No correlation was detected between the biofilm phenotype pattern and the RND efflux pump gene expression in biofilm cells relative to planktonic cells. In conclusion, the role of the RND efflux pumps AdeABC, AdeFGH, and AdeIJK in biofilm formation does not appear to be pivotal and the expression differs according to the genetic background of each strain. Thus, these pumps may not be a promising target for biofilm inhibition.

## 1. Introduction

*Acinetobacter baumannii* (*A. baumannii)* has emerged globally as an opportunistic multidrug-resistant (MDR) pathogen that is primarily linked with healthcare-associated infections, including pneumonia, meningitis, bacteremia and urinary tract infections [1]. *A. baumannii* is characterized by its natural competence that makes it more capable of incorporating foreign DNA, which can subsequently increase its adaptability to hostile environments [2,3]. The emergence of resistant isolates to widely prescribed antimicrobial agents leads to a high rate of treatment failure, and a higher mortality rate for infections caused by *Acinetobacter* spp. The poor prognosis of infections caused by *A. baumannii* puts this bacterium among the list of “top critical threat” pathogens [4]. Various mechanisms are associated with the multidrug resistance phenotype, including enzymatic degradation, alteration of a target site, decreased membrane permeability, biofilm formation, and increased expression of the efflux pumps [5]. It was reported that the efflux pumps play a dual role in antibiotic resistance either directly by extruding antibiotics or indirectly by biofilm formation. Hence, the correlation between the wide substrate specificity efflux pump systems and the bacterial ability to form biofilms has become an important area to explore [6].

The efflux pumps implicated in bacterial multidrug resistance perform a variety of roles in the transition of planktonic cells to biofilms. These pumps actively extrude quorum-sensing auto-inducers, as well as antimicrobial agents and metabolic intermediates, resulting in direct and indirect regulation of biofilm formation and quorum sensing [7]. Some pumps also cause change in cell membrane composition, thus, altering biofilm formation [8].

*A. baumannii* are characterized by six major efflux pump families, which are major facilitator superfamily (MFS), multidrug and toxic efflux (MATE), resistance nodulation-division (RND), small multidrug resistance (SMR), ATP binding cassette (ABC) [9], and the novel proteobacterial antimicrobial compound efflux (PACE) family [10]. Among the efflux systems, RND pumps are the most prevalent systems in Gram-negative bacteria [11]. RND efflux system extrudes a wide range of substrates that are structurally unrelated [12]. Three *Acinetobacter* drug efflux pumps (Ade) belong to the RND systems, namely AdeABC, AdeFGH, and AdeIJK, which have been widely distributed in *A. baumannii* [13]. In addition, these efflux pumps have common substrates, which are fluoroquinolones and chloramphenicol [11]. Little is known regarding the association between efflux pumps in *A. baumannii* and biofilm formation, hence, the aim of our study is to investigate the role of the RND efflux pump system in biofilm formation by *A. baumannii*. This can open new avenues for the treatment of biofilm-related infections.

## 2. Results

### 2.1. Biofilm Formation Ability of A. baumannii Isolates and Enterobacterial Repetitive Intergenic Consensus (ERIC) Genotyping

Thirty-four MDR *A. baumannii* isolates (collected and genotyped by ERIC typing) were grouped into 9 ERIC clusters (A to I). The biofilm formation index (BFI) of the tested isolates was classified as strong, moderate, weak, and non-adherent, as in Table 1. Thus, eight of the isolates were classified as strong (8/34; 23.5%), ten as moderate (11/34; 32.3%), eleven as weak (11/34; 32.3%), and five as non-adherent (4/34; 11.7%) (Table 1). The standard strain showed strong biofilm forming ability (BFI = 1.7). Each ERIC cluster was found to include isolates with the same biofilm phenotype except clusters G and H, which included isolates with different biofilm phenotypes (Table 1).

### 2.2. The Effect of Efflux Pump Substrate and Efflux Pump Inhibitor on Biofilm Phenotype by A. baumannii Isolates

#### 2.2.1. Efflux Pump Substrate (Levofloxacin)

Levofloxacin-resistant isolates represented (25/34; 73.5%) of the isolates as confirmed by the high MICs of levofloxacin ranging from 4 µg/mL to 216 µg/mL. In order to determine the role of the efflux pump in levofloxacin resistance, CCCP was used at a concentration of 12.5 µg/mL, which does not reduce the growth of isolates as reflected by their OD_600_, compared to control untreated culture. The addition of CCCP to the bacterial culture of levofloxacin-resistant isolates caused a reduction in MIC by four folds or more in 56% (14/25) of resistant isolates as shown in Table 2. On the other hand, 40% (10/25) of levofloxacin-resistant isolates became sensitive upon treatment with CCCP (Table 2).

**Table 2:** Change in the phenotypic resistance pattern of isolates to levofloxacin under the effect of the efflux pump inhibitor carbonyl cyanide 3-chlorophenylhydrazone (CCCP) (*n* = 25).

Isolate Number	MIC (µg/mL)	MIC + CCCP (µg/mL)	Phenotypic Factor (F) ^#^ CCCP	Resistance Phenotype ^†^ (−/+ CCCP)
***A. baumannii* ATCC 19606**	1	1	1	S/S
**4**	64	32	2	R/R
**7**	8	4	2	R/R
**12**	8	4	2	R/R
**14**	64	32	2	R/R
**15**	8	4	2	R/R
**22**	4	1	4 *	R/S
**23**	8	1	8 *	R/S
**24**	64	1	64 *	R/S
**25**	8	1	8 *	R/S
**26**	64	1	64 *	R/S
**27**	4	1	4 *	R/S
**28**	256	128	2	R/R
**29**	256	128	2	R/R
**30**	256	1	256 *	R/S
**32**	64	8	8 *	R/R
**33**	16	4	4 *	R/R
**35**	32	8	4 *	R/R
**36**	32	1	32 *	R/S
**37**	32	16	2	R/R
**39**	8	1	8 *	R/S
**40**	64	32	2	R/R
**41**	128	32	4 *	R/R
**43**	4	2	2	R/I
**44**	32	16	2	R/R
**45**	16	2	8 *	R/I

^†^ S: Susceptible (MIC ≤ 1 µg/mL), I: Intermediate (MIC = 2 µg/mL), R: Resistant (MIC ≥ 4µg/mL). ^#^ Phenotypic efflux factor (F) = MIC without CCCP (EPI)/MIC with CCCP (EPI); * Isolates showing > 4 phenotypic efflux factor with CCCP 12.5 µg/mL.

After determining the MIC of levofloxacin against isolates, the effect of increased efflux pump activity by sub-MICs of levofloxacin on biofilm formation was studied. The addition of sub-MICs of levofloxacin to bacterial culture significantly increased (*p* < 0.05) the biofilm formation pattern of 20.5% (7/34) of the isolates (Table 3). No correlation was detected between fold change in the BFI using sub-MIC of levofloxacin and the biofilm formation potential of the isolates (Spearman’s *r_s_* = −0.47, *p* > 0.05).

#### 2.2.2. Efflux Pump Inhibitor (CCCP)

Using CCCP for efflux pump inhibition significantly reduced (*p* < 0.05) biofilm formation in 17.6% (6/34) of the isolates (Table 3). However, isolates showing change in biofilm formation potential in the presence of CCCP were different from those affected by sub-MICs of levofloxacin. No correlation was detected between fold change in BFI using CCCP and the biofilm formation potential of the isolates (Spearman’s *r_s_* = 0.07, *p* > 0.05).

### 2.3. Expression Levels of the adeB, adeG, and adeJ Efflux Pump Genes in Planktonic and Biofilm Cells Using Quantitative Real-Time PCR (qRT-PCR)

**Figure 1:**
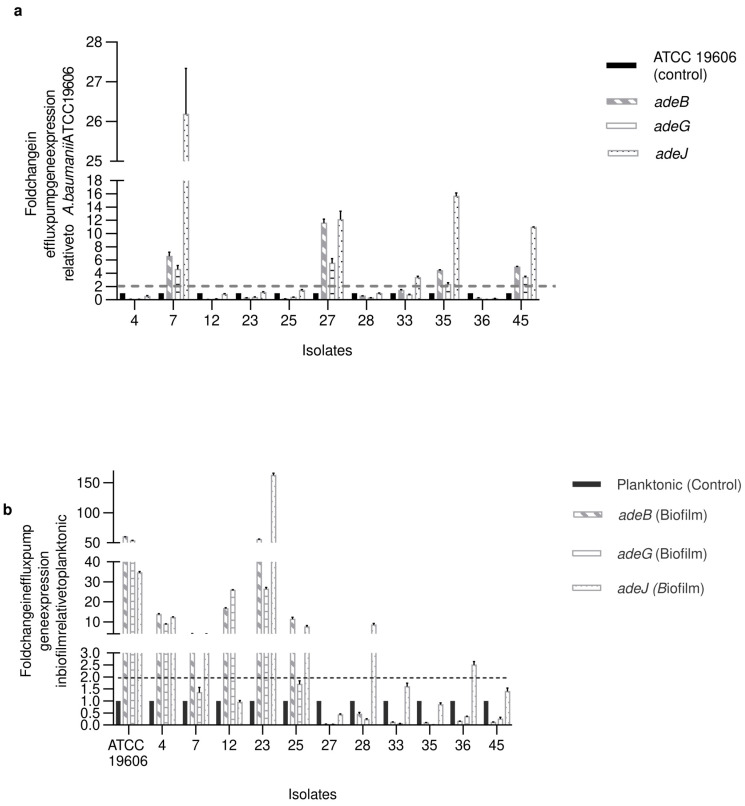
Fold changes in expression of the *adeB*, *adeG*, and *adeJ* genes in *A. baumannii* isolates. The bars represent relative expression levels of each gene with the corresponding control. (**a**) Expression of isolates in planktonic cells relative to standard strain (ATCC 19606) (control); (**b**) Expression of isolates in biofilm forming cells and standard strain relative to their planktonic cells (control). The error bars represent the standard error of mean (SEM). All values are means ± SEM. 16S rRNA was used as housekeeping gene control. Strong biofilm forming isolate (Standard strain ATCC 19606, 4, 23, 25, 28), moderate biofilm forming isolate (27, 33, 35, 45), weak biofilm forming (7, 12), and non-adherent isolate (36). Dotted line: Change in expression > 2 folds.

The three efflux genes were detected by conventional PCR in the thirty-four isolates and the standard *A. baumannii* strain (ATCC 19606). Gene expression analysis was done on eleven isolates and a standard strain by qRT-PCR analysis to measure the expression of the three RND efflux pumps. Isolates for gene expression were selected based on the ERIC cluster typing by selecting an isolate from each cluster that represents the biofilm phenotype in this group. A representative isolate was selected from each ERIC cluster except for clusters G and H, which comprise two biofilm phenotypes, therefore, two isolates were selected from these groups. Measuring the expression levels of *adeB*, *adeG*, and *adeJ* genes showed overexpression in five out of the eleven tested isolates relative to the standard strain (Figure 1a). Isolates with overexpression in the efflux pump were moderate (isolates no. 27, 33, 35, 45) and weak biofilm forming (isolate no. 7). It was observed that the *adeJ* gene relative expression was remarkably higher than the other two RND efflux pump genes in tested isolates.

The relative expression pattern of *adeB*, *adeG*, and *adeJ* varied among different isolates with different biofilm forming abilities. Five strong biofilm forming isolates (Standard strain ATCC 19606, 4, 23, 25, and 28) showed more than a twofold increase in the gene expression of efflux pumps in biofilm form than planktonic form, while the remaining strong and moderate biofilm isolates (27, 33, 35, 45) showed either more than a twofold reduction or no change in gene expression of the same efflux pumps compared to planktonic forms (Figure 1b). No correlation was detected between the biofilm formation ability of isolates and the relative expression of the RND efflux pump genes (Spearman’s *r_s_* = 0.17, 0.14, and 0.35 for *adeB*, *adeG*, and *adeJ*, respectively; *p* > 0.05). Isolates that belong to the same ERIC clusters (G:28–36 and H:4–23) or closely related clusters (A:25 and B:7) showed similar efflux pump relative expression patterns despite the difference in biofilm phenotype in some cases.

## 3. Discussion

*A. baumannii* has become a major therapeutic problem worldwide due to its high prevalence in hospital environments and acquisition of antimicrobial resistance [6]. *A. baumannii* has developed different mechanisms to resist antibiotics and among them is extrusion of toxic compounds by efflux pumps and biofilm formation [14]. The resistance nodulation division (RND) efflux pump family represents the major type of efflux pump in Gram negative bacteria [15]. Therefore, it has been the focus of our current study. Evaluating the biofilm formation potential of the MDR *A. baumannii* isolates in our study revealed that, overall, 88.2% (30/34) of isolates were biofilm producers with variable phenotypes that ranged from strong to weak. The presence of weak and non-adherent biofilm isolates representing 44.1% of the MDR isolates indicates that biofilm formation has no association with antibiotic resistance as reported by Chen and colleagues [13].

Recently, the role of efflux pumps in biofilm formation has been the focus of different studies seeking alternatives to antimicrobials in controlling biofilm formation [6,16]. To study the effect of the efflux pump substrate and efflux pump inhibitors on biofilm phenotypes, we utilized sub-MICs of levofloxacin and CCCP, respectively. Levofloxacin is a common substrate for the three RND efflux pumps: AdeABC, AdeFGH, and AdeIJK [17,18].

In the present study, the resistance of *A. baumannii* isolates to levofloxacin was detected in 73.5% (25/34) of the isolates, a percentage that is comparable to findings of a previous study by Zaki and colleagues [19] but less than resistance percentages detected in other studies that showed resistance rates of 85–92% [20,21]. The susceptibility of 56% (14/25) of strains to levofloxacin has increased in the presence of the efflux pump inhibitor CCCP; however, only 40% of resistant isolates have restored susceptibility under the effect of CCCP. Similar reduction in quinolone resistance after the addition of CCCP was reported in previous studies [22,23], indicating the involvement of efflux system in these isolates. The failure of CCCP in restoring susceptibility to quinolone in some isolates can indicate the presence of more than one mechanism of resistance to levofloxacin in these isolates, such as mutations in the *gyrA* or *parC* genes encoding gyrase subunit A and topoisomerase IV subunit C, respectively [24]. The addition of sub-MICs of levofloxacin (efflux pump substrate) to bacterial culture significantly increased the biofilm formation pattern (*p* < 0.05) in 20.5% (7/34) of isolates. A study by He and colleagues [25] illustrated that sub-MIC of levofloxacin caused overexpression of the *adeFGH* gene, resulting in accelerated synthesis and transport of quorum sensing molecules during biofilm formation. On the other hand, efflux pump inhibition by CCCP significantly reduced (*p* < 0.05) biofilm formation in 17.6% (6/34) of isolates. Strains affected by levofloxacin were different from those affected by CCCP indicating that the efflux pumps impacted in each of the two cases were different [26]. Moreover, the exposure of *A. baumannii* to sub-inhibitory concentrations of antimicrobials did not only increase substrate efflux by the efflux pump but also changed the expression of genes controlling biofilm formation, including genes regulating pili, other efflux pumps, and virulence factors [27].

The RND efflux pump genes were detected in all tested isolates. Likewise, in another study, AdeABC, AdeFGH, and AdeIJK pumps were detected in 92.18%, 98.43%, and 89.06% of strains, respectively [28]. The relative gene expression of the major part of tripartite efflux systems (*adeB*, *adeG,* and *adeJ*) was measured by qPCR for eleven isolates representing different ERIC clusters relative to that of the reference *A. baumannii* strain (ATCC 19606). CCCP increased the levofloxacin susceptibility of the five isolates that did not show overexpression for any of the efflux pumps. No correlation was detected between the effect of CCCP on resistance to levofloxacin as indicated by a reduction in the MIC and the RND efflux pump expression (Spearman’s *r_s_* = −0.08, −0.16, and −0.02, for *adeB*, *adeG*, and *adeJ,* respectively; *p* > 0.05). This result indicates the involvement of other efflux pumps in levofloxacin resistance by these isolates, such as *abeM* and *abaQ*, that belong to the MATE and MFS efflux systems, respectively [29]. The previous efflux pumps can also be inhibited by CCCP [30,31].

Previous studies on the role of *A. baumannii* efflux pumps in biofilm formation were based on knocking out the RND efflux pump genes in a limited number of strains and then measuring the impact of this on biofilm formation [16,32,33] or on measuring the relative expression of efflux genes in the planktonic form and correlating the expression levels to the degree of biofilm formation [6,13]. In our assessment, these previous approaches are not adequate or even representative for the bacteria, given its distinct phenotypic and genotypic characteristics. Moreover, these methods do not reflect the actual expression pattern in the biofilm form. Thus, the expression of *adeB, adeG*, and *adeJ* efflux pump genes in the biofilm form relative to the planktonic form was studied to illustrate the role of these pumps in the mature biofilm of *A. baumannii*. Results have shown that the relative expression pattern of the RND pumps genes varied among different isolates with different biofilm forming abilities. The lack of correlation between efflux pump gene expression and biofilm formation potential suggests that the RND efflux pump role in biofilm formation is not pivotal. Some studies reported the lack of association between the activity of the efflux pump family and biofilm formation [6,13,28]. Other studies reported a negative correlation between the activity of one or more of the RND efflux pump genes *adeB, adeG*, and *adeJ* and biofilm formation [6,12,16,32,34,35,36]. The negative correlation observation was explained by the presence of associated changes in the bacterial membrane with efflux pump overexpression, such as the reduction in *Csu-pili* and the downregulation of genes involved in iron acquisition and motility [6,16,33]. Furthermore, other studies reported that the increase in expression of one or more of the *adeB*, *adeG,* and *adeJ* genes was associated with increased biofilm formation [6,16,25,28,33,36]. The overexpression of the *adeFGH* efflux pump gene was suggested to play a role in the synthesis and efflux of quorum sensing molecules during biofilm formation [25,37].

Other explanations highlighted operons as the controllers of these pumps through controlling other genes contributing to biofilm formation. In this scenario, the efflux pumps are not directly in control of the biofilm phenotype. For example, the low expression of *adeABC* is associated with the reduced expression of other genes involved in biofilm formation, motility, and virulence because these genes are subjected to control by the regulator adeRS [33]. The global regulator *adeN* of the *adeIJK* does not only regulate the *adeIJK* expression but also the expression of many other factors involved in biofilm formation [38]. In addition, the levels of guanosine tetraphosphate (ppGpp) could control the expression of *adeABC* and *adeIJK* [39,40]. Another controller for the expression of the *adeIJK* and *adeABC* efflux pump genes is the BaeSR (two component system), which is involved in controlling the expression of other efflux pumps that favor biofilm formation (such as MacAB-TolC) through cell detoxification and maintenance responses [39,41].

In previous studies, different *A. baumannii* strains exhibited different responses to RND efflux pump gene deletion and the role of these pumps in biofilm formation may differ between the strains [6,16,33]. *A. baumannii* was shown to require certain expression profiles of efflux pumps to initiate and maintain biofilm formation [33]. Furthermore, the overexpression profile of the RND pumps could be specific for each country [8]. In our study, it was observed that the *adeJ* gene relative expression was remarkably higher than the two other RND efflux pump genes in tested isolates. Similarly, in French and Korean studies, overexpression of the *adeJ* gene was detected as compared to the expression of other RND pumps [6,29]. Overexpression of *adeG* was reported in isolates from Canada [42].

Amin and colleagues reported that isolates that are genotypically characterized according to ERIC typing showed similar biofilm formation potential [28]. Similarly, we observed that isolates that belong to the same ERIC clone showed similar biofilm forming potential, except in clusters B and G. Notwithstanding, isolates that belong to the same ERIC clusters (G:28–36 and H:4–23) or closely related clusters (A:25 and B:7) showed similar efflux pump relative expression patterns in biofilm form compared to the planktonic form despite the difference in the biofilm phenotype.

In conclusion, overexpression of the RND efflux pumps AdeABC, AdeFGH, and AdeIJK was not correlated with the biofilm phenotype in any of the tested isolates. Therefore, their roles in biofilm formation do not appear to be pivotal and their expression differs according to the genetic background of each strain. Consequently, efflux pumps may be a part of more complicated mechanisms that contribute to biofilm formation.

## 4. Materials and Methods

### 4.1. Bacterial Isolates 

Thirty-four Multidrug Resistant (MDR) *A. baumannii* isolates from blood samples of cancer patients at the National Cancer Institute (NCI) from our previous study were genotypically characterized via enterobacterial repetitive intergenic consensus (ERIC)–PCR [2]. Approval was obtained from the ethics committee of the October University for Modern Sciences and Arts. Standard *A. baumannii* (ATCC 19606) was used as a control for the gene expression assay.

### 4.2. Assessment of the Biofilm Forming Ability of Isolates

Crystal violet assays were used to determine the biofilm forming ability of the isolates [43]. The turbidity of an overnight culture was adjusted to the equivalent of McFarland 0.5, and 200 µL of the adjusted culture was added to each well of a 96-well microtiter plate. After overnight incubation at 37 °C, growth turbidity was measured at 600 nm (OD_growth_), then the biofilm was stained by crystal violet (2% *w*/*v*). Plates were left for air drying before destaining with glacial acetic acid (33% *v*/*v*). Finally, biofilm biomass was measured using a microplate reader at 545 nm (OD_CV_). Biofilm formation was evaluated using a biofilm formation index [BFI]: (OD_CV_ Biofilm-OD_CV_ Control)/OD_growth_. The biofilm formation ability of the isolates was classified into strong, moderate, weak, and non-adherent according to the BFI values described by Yaikhan and colleagues [44].

### 4.3. Evaluation of the Role of Efflux Pumps on Biofilm Formation Phenotypes

The efflux pump inhibitor (EPI) named carbonyl cyanide 3-chlorophenylhydrazone (CCCP) (ChemCruz, Dallas, Texas, USA) was used to phenotypically evaluate the role of efflux pumps in biofilm formation and levofloxacin resistance. The effects of two commonly used concentrations of CCCP (12.5 µg/mL and 25 µg/mL) were tested on bacterial growth. The concentrations that did not reduce growth significantly as determined spectrophotometrically at OD_600_, compared to control untreated culture, were selected for efflux pump inhibition tests [29].

Efflux pump substrate was used to study the effect of efflux pump activation on biofilm formation. Levofloxacin is a common substrate for the three RND efflux pumps under study, and the role of this substrate on the pump activity was studied at sub-MIC levels. Firstly, the MICs of levofloxacin were determined in addition to the change in MICs in the presence of CCCP. A calculated phenotypic efflux factor F (MIC without CCCP (EPI) /MIC with CCCP (EPI)) of 4 and above reflects the role of efflux pumps in levofloxacin resistance [45]. Secondly, the effect of efflux pump on biofilm phenotype was determined by assessing the difference in BFI in presence of CCCP and sub-MICs of levofloxacin which are used as EPI and efflux pump substrate, respectively. Fold change in biofilm = BFI of untreated isolate/BFI in presence of CCCP or sub-MIC of levofloxacin [25,46].

### 4.4. Detection of the Efflux Pump Genes adeABC, adeFGH, and adeIJK

Pure colonies of *A. baumannii* isolates were used for DNA extraction using the Thermo Scientific™ GeneJet™ genomic DNA purification kit (Thermo Scientific, Waltham, MA, USA), in accordance with the manufacturer’s recommendations, and then kept at −20 °C. The genes *adeB, adeG,* and *adeJ* were selected for amplification. These genes were selected because they represent an essential part of the Ade efflux system. PCR was performed to screen for the presence of the efflux transporter genes *AdeB, AdeG*, and *AdeJ*, using the primers and annealing temperatures described in Table 4. Genes were amplified by initial denaturation at 94 °C for 5 min, followed by 35 cycles consisting of 3 phases: DNA denaturation at 94 °C for 0.5 min, annealing according to Table 4 for 0.5 min, elongation at 72 °C for 0.5 min, and a final elongation at 72 °C for 10 min.

**Table 4:** Oligonucleotides sequences and annealing temperatures.

Primer	Sequence 5′—3′	Annealing Temperature (°C)	Ampilicon Size bp	Reference
*adeB*	F: GCAGAGCGTACTCGGAATGT R:CCACTGAAACCCCATCCCAA	57	101	[47]
*adeG*	F:GCGTTGCTGTGACAGATGTT R:TTGTGCACGGACCTGATAAA	52	104	[5]
*adeJ*	F:TTCGGTGGCTCATACGCAAT R:GGAGCACCACCTAACTGACC	57	137	[47]
*16s rRNA*	F:AGCTAACGCGATAAGTAGACCG R:TGTCAAGGCCAGGTAAGGTTC	57	137	[47]

### 4.5. Extraction of Total Bacterial RNA from Planktonic and Biofilm Forming Cells

A representative isolate from each ERIC cluster was selected for gene expression analysis by the Quantitative polymerase chain reaction (qPCR). Isolates were grown overnight at 37 °C in Lauria Bertani (LB) broth and diluted to McFarland 0.5. A volume of 7 mL of adjusted suspension from each isolate was added in each well of 6-well plates (Greiner Bio-One, Kremsmünster, Austria) and was incubated at 37 °C for 24 h. After incubation, culture suspension was removed from wells for extracting RNA from planktonic bacteria. Cells adhering to the plate wells were washed twice by cold sterile Phosphate buffer saline (PBS) and, then, dislodged by sonication before suspension in falcon tube containing PBS. Total RNA was isolated from planktonic and adherent cells using RNeasy^®^ Minikit (Qiagen, Hilden, Germany) according to the manufacturer’s instructions. Contaminating DNA was removed by RNase-free DNase I (New England Biolabs, USA). The concentrations and purity of RNA were quantified with a NanoDrop^TM^ Spectrophotometer (ThermoScientific^TM^, Ipswich, MA, USA) at 260 and 280 nm (260/280 ratio of >1.8) and agarose gel electrophoresis of RNA samples verified its integrity. Finally, the RevertAid First strand cDNA synthesis kit (ThermoScientific^TM^, Waltham, MA, USA) was used to reverse transcribe 1 μg of total RNA sample into cDNA.

### 4.6. Quantification of the Expression of the RND Efflux Pump Genes (adeB, adeG, adeJ) in Biofilm and Planktonic Forms Using RT-qPCR

Real-time quantitative PCR was used to measure the relative expression of the RND-family efflux pump genes (*adeB, adeG, adeJ*) using Maxima SYBR Green qPCR Master Mix with ROX (ThermoScientific, Waltham, Massachusetts, USA) and primers listed in Table 4. The expression of pump genes was normalized to the housekeeping gene (16S rRNA). Expression quantification was done using the StepOnePlus^TM^ Real-Time PCR System and software (Applied Biosystems, Waltham, Massachusetts, USA) according to the manufacturer’s instructions. After a 10 min activation of the modified Taq polymerase at 95 °C, 40 cycles of 15 s at 95 °C, 30 s at 60 °C, and 30 s at 72 °C were performed. Data acquisition was done at 60 °C and 72 °C. The relative gene expression for pump genes transcripts was calculated against internal control 16S rRNA gene. The relative expression of efflux pump genes in the tested isolates was calculated against those of *A. baumannii* ATCC 19606 (expression = 1), which served as the control to determine overexpressed pump genes. The expression of the efflux pump genes in the biofilm form was determined relative to their expression in the planktonic form. The 2^−ΔΔCT^ method was used to calculate the relative expression level of pump genes. A significant effect on gene expression was concluded when the corresponding ratios were >2.0. All reactions were performed in triplicate [24].

### 4.7. Statistical Analysis

We used the Prism version 8.3.0 software for Windows (GraphPad Software) for data analysis and graph plotting. The relationships between different factors were assessed by calculating Spearman’s correlation coefficients. The student’s t-test was used for data analysis of the gene expression. Statistical differences were considered significant at *p* < 0.05.

## Figures and Tables

**Table 1:** Biofilm formation phenotypes of *Acinetobacter baumannii* isolates genotypically characterized by ERIC-PCR.

ERIC Cluster Group	Isolate Number	Biofilm Formation Pattern
**A**	25	Strong
	30	Strong
**B**	7	Weak
	9	Weak
	37	Weak
**C**	22	Strong
	27	Moderate
	39	Strong
	43	Strong
**D**	1	Moderate
	26	Moderate
	44	Moderate
	45	Moderate
	47	Moderate
**E**	32	Moderate
	33	Moderate
	46	Moderate
**F**	31	Moderate
	35	Moderate
**G**	3	Weak
	6	Weak
	24	Non-Adherent
	28	Strong
	29	Weak
	36	Non-Adherent
	40	Non-Adherent
	41	Non-Adherent
**H**	2	Weak
	4	Strong
	14	Weak
	15	Weak
	23	Strong
**I**	12	Weak
	21	Weak

**Table 3:** Biofilm formation potential by *Acinetobacter baumannii* isolates and the effect of efflux pump inhibitor (CCCP) and efflux pump substrate (levofloxacin at 0.25x MIC) on biofilm formation.

ERIC Cluster Group	Isolate Number	BFI ^#^	Biofilm Formation Pattern	BFI with Sub-MIC of Levofloxacin (Fold Change)	BFI with CCCP (Fold Change)
**A**	25 ^#^	2.60	Strong	2.30(0.91)	2.20 (0.88)
	30	1.30	Strong	1.24 (0.92)	1.04 (0.80)
**B**	7 ^#^	0.70	Weak	0.95 (1.35) *	0.70 (1.00)
	9	0.70	Weak	0.73 (1.05)	0.80 (1.17)
	37	0.60	Weak	0.54 (0.90)	0.61 (1.02)
**C**	22	1.27	Strong	1.10 (0.90)	1.20 (0.94)
	27 ^#^	2.37	Moderate	2.48 (1.05)	2.08 (0.88) *
	39	1.43	Strong	1.37 (0.89)	1.43 (1.00)
	43	1.10	Strong	0.99 (0.90)	1.10 (0.90) *
**D**	1	0.80	Moderate	0.84 (1.05)	0.90 (1.12)
	26	0.70	Moderate	0.88 (1.26) *	1.24 (1.77)
	44	0.90	Moderate	1.80 (2.00) **	0.85 (0.95)
	45 ^#^	0.79	Moderate	0.86 (1.08)	0.66 (0.83) *
	47	0.90	Moderate	1.00 (1.10)	0.80 (0.88)
**E**	32	0.95	Moderate	0.90 (0.94)	0.95 (1.00)
	33 ^#^	0.96	Moderate	0.86 (0.90)	0.96 (1.00)
	46	0.80	Moderate	0.90 (1.12)	0.85 (1.06)
**F**	31	0.90	Moderate	0.95 (1.05)	1.00 (1.10)
	35 ^#^	1.68	Moderate	1.39 (0.83)	1.30 (0.78)
**G**	3	0.65	Weak	0.70 (1.07)	0.80 (1.23)
	6	0.60	Weak	0.65 (1.08)	0.58 (0.96)
	24	0.29	Non-Adherent	0.28 (0.99)	0.49 (1.70)
	28 ^#^	1.50	Strong	1.48 (0.98)	1.29 (0.86) *
	29	0.62	Weak	0.56 (0.90)	0.62 (1.00)
	36 ^#^	0.29	Non-Adherent	0.46 (1.30) *	0.29 (1.00)
	40	0.04	Non-Adherent	0.19 (5.00) *	0.035 (0.90)
	41	0.11	Non-Adherent	0.27 (2.50) **	0.09 (0.90)
**H**	2	0.60	Weak	0.64 (1.08)	0.67 (1.12)
	4 ^#^	2.00	Strong	1.78 (0.89)	2.34 (1.17)
	14	0.55	Weak	0.48 (0.89)	0.42 (0.76)
	15	0.59	Weak	0.87 (1.49) *	0.67 (0.40) *
	23 ^#^	2.40	Strong	2.16 (0.90)	0.79 (0.33) *
**I**	12 ^#^	0.52	Weak	0.58 (1.13)	0.42 (0.80)
	21	0.50	Weak	0.60 (1.20)	0.55 (1.10)

BFI: ≥1.1 = strong, 0.7–1.09 = moderate, 0.35–0.69 = weak, <0.35 = non adherent. ^#^ Isolates selected for gene expression analysis by qPCR. Statistical analysis by unpaired t-test, with significance * *p* < 0.05, ** *p* < 0.01.

## Data Availability

All relevant data are included within the manuscript.

## References

[1] Kim H.-R., Shin D.-S., Jang H.-I., Eom Y.-B. (2020). Anti-biofilm and anti-virulence effects of zerumbone against *Acinetobacter baumannii*. Microbiology.

[2] Wasfi R., Rasslan F., Hassan S.S., Ashour H.M., El-Rahman O.A.A. (2021). Co-Existence of Carbapenemase-Encoding Genes in *Acinetobacter baumannii* from Cancer Patients. Infect. Dis. Ther..

[3] Zafer M.M., Hussein A.F.A., Al-Agamy M.H., Radwan H.H., Hamed S.M. (2021). Genomic Characterization of Extensively Drug-Resistant NDM-Producing *Acinetobacter baumannii* Clinical Isolates With the Emergence of Novel blaADC-257. Front. Microbiol..

[4] Kengkla K., Kongpakwattana K., Saokaew S., Apisarnthanarak A., Chaiyakunapruk N. (2018). Comparative efficacy and safety of treatment options for MDR and XDR *Acinetobacter baumannii* infections: A systematic review and network meta-analysis. J. Antimicrob. Chemother..

[5] Nowak J., Seifert H., Higgins P. (2015). Prevalence of eight resistance-nodulation-division efflux pump genes in epidemiologically characterized *Acinetobacter baumannii* of worldwide origin. J. Med. Microbiol..

[6] Kim C.-M., Park G., Ko Y.J., Kang S.-H., Jang S.J. (2021). Relationships between Relative Expression of RND Efflux Pump Genes, H33342 Efflux Activity, Biofilm-Forming Activity, and Antimicrobial Resistance in *Acinetobacter baumannii* Clinical Isolates. Jpn. J. Infect. Dis..

[7] Navidifar T., Amin M., Rashno M. (2019). Effects of sub-inhibitory concentrations of meropenem and tigecycline on the expression of genes regulating pili, efflux pumps and virulence factors involved in biofilm formation by *Acinetobacter baumannii*. Infect. Drug Resist..

[8] Kornelsen V., Kumar A. (2021). Update on Multidrug Resistance Efflux Pumps in *Acinetobacter* spp.. Antimicrob. Agents Chemother..

[9] Abdi S.N., Ghotaslou R., Ganbarov K., Mobed A., Tanomand A., Yousefi M., Asgharzadeh M., Kafil H.S. (2020). *Acinetobacter baumannii* Efflux Pumps and Antibiotic Resistance. Infect. Drug Resist..

[10] Hassan K.A., Liu Q., Elbourne L.D., Ahmad I., Sharples D., Naidu V., Chan C.L., Li L., Harborne S.P., Pokhrel A. (2018). Pacing across the membrane: The novel PACE family of efflux pumps is widespread in Gram-negative pathogens. Res. Microbiol..

[11] Barnie P.A., Xing L., Su Z., Xu H. (2014). Development of Efflux Pumps and Inhibitors (EPIs) in *A. baumanii*. Clin. Microbiol. Open Access.

[12] Yoon E.-J., Chabane Y.N., Goussard S., Snesrud E., Courvalin P., Dé E., Grillot-Courvalin C. (2015). Contribution of Resistance-Nodulation-Cell Division Efflux Systems to Antibiotic Resistance and Biofilm Formation in *Acinetobacter baumannii*. Mbio.

[13] Chen L., Li H., Wen H., Zhao B., Niu Y., Mo Q., Wu Y. (2020). Biofilm formation in *Acinetobacter baumannii* was inhibited by PAβN while it had no association with antibiotic resistance. Microbiologyopen.

[14] Singh H., Thangaraj P., Chakrabarti A. (2013). *Acinetobacter baumannii*: A Brief Account of Mechanisms of Multidrug Resistance and Current and Future Therapeutic Management. J. Clin. Diagn. Res..

[15] Nikaido H., Takatsuka Y. (2009). Mechanisms of RND multidrug efflux pumps. Biochim. Biophys. Acta.

[16] Leus I.V., Adamiak J., Trinh A.N., Smith R.D., Smith L., Richardson S., Ernst R.K., Zgurskaya H.I. (2020). Inactivation of AdeABC and AdeIJK efflux pumps elicits specific nonoverlapping transcriptional and phenotypic responses in *Acinetobacter baumannii*. Mol. Microbiol..

[17] Damier-Piolle L., Magnet S., Brémont S., Lambert T., Courvalin P. (2008). AdeIJK, a Resistance-Nodulation-Cell Division Pump Effluxing Multiple Antibiotics in *Acinetobacter baumannii*. Antimicrob. Agents Chemother..

[18] Coyne S., Guigon G., Courvalin P., Périchon B. (2010). Screening and Quantification of the Expression of Antibiotic Resistance Genes in *Acinetobacter baumannii* with a Microarray. Antimicrob. Agents Chemother..

[19] Zaki M.E.S., ElKheir N.A., Mofreh M. (2018). Molecular Study of Quinolone Resistance Determining Regions of gyrA Gene and parC Genes in Clinical Isolates of *Acintobacter baumannii* Resistant to Fluoroquinolone. Open Microbiol. J..

[20] Alkasaby N.M., Zaki M.E.S. (2017). Molecular Study of *Acinetobacter baumannii* Isolates for Metallo-*β*-Lactamases and Extended-Spectrum-*β*-Lactamases Genes in Intensive Care Unit, Mansoura University Hospital, Egypt. Int. J. Microbiol..

[21] Al-Agamy M.H., Khalaf N.G., Tawfick M.M., Shibl A.M., Kholy A.E. (2014). Molecular characterization of carbapenem-insensitive *Acinetobacter baumannii* in Egypt. Int. J. Infect. Dis..

[22] Sekyere J.O., Amoako D.G. (2017). Carbonyl Cyanide m-Chlorophenylhydrazine (CCCP) Reverses Resistance to Colistin, but Not to Carbapenems and Tigecycline in Multidrug-Resistant Enterobacteriaceae. Front. Microbiol..

[23] Lin L., Ling B.-D., Li X.-Z. (2009). Distribution of the multidrug efflux pump genes, adeABC, adeDE and adeIJK, and class 1 integron genes in multiple-antimicrobial-resistant clinical isolates of *Acinetobacter baumannii*–Acinetobacter calcoaceticus complex. Int. J. Antimicrob. Agents.

[24] Roy S., Chatterjee S., Bhattacharjee A., Chattopadhyay P., Saha B., Dutta S., Basu S. (2021). Overexpression of Efflux Pumps, Mutations in the Pumps’ Regulators, Chromosomal Mutations, and AAC(6′)-Ib-cr Are Associated with Fluoroquinolone Resistance in Diverse Sequence Types of Neonatal Septicaemic *Acinetobacter baumannii*: A 7-Year Single Center Study. Front. Microbiol..

[25] He X., Lu F., Yuan F., Jiang D., Zhao P., Zhu J., Cheng H., Cao J., Lu G. (2015). Biofilm Formation Caused by Clinical *Acinetobacter baumannii* Isolates Is Associated with Overexpression of the AdeFGH Efflux Pump. Antimicrob. Agents Chemother..

[26] Poole K. (2002). Outer Membranes and Efflux: The Path to Multidrug Resistance in Gram—Negative Bacteria. Curr. Pharm. Biotechnol..

[27] Gallagher P., Baker S. (2020). Developing new therapeutic approaches for treating infections caused by multi-drug resistant *Acinetobacter baumannii*: *Acinetobacter baumannii* therapeutics. J. Infect..

[28] Amin M., Navidifar T., Shooshtari F.S., Rashno M., Savari M., Jahangirmehr F., Arshadi M. (2019). Association Between Biofilm Formation, Structure, and the Expression Levels of Genes Related to biofilm formation and Biofilm-Specific Resistance of *Acinetobacter baumannii* Strains Isolated from Burn Infection in Ahvaz, Iran. Infect. Drug Resist..

[29] Choquet M., Lohou E., Pair E., Sonnet P., Mullié C. (2021). Efflux Pump Overexpression Profiling in *Acinetobacter baumannii* and Study of New 1-(1-Naphthylmethyl)-Piperazine Analogs as Potential Efflux Inhibitors. Antimicrob. Agents Chemother..

[30] Pérez-Varela M., Corral J., Aranda J., Barbé J. (2018). Functional Characterization of AbaQ, a Novel Efflux Pump Mediating Quinolone Resistance in *Acinetobacter baumannii*. Antimicrob. Agents Chemother..

[31] Su X.-Z., Chen J., Mizushima T., Kuroda T., Tsuchiya T. (2005). AbeM, an H ^+^ -Coupled *Acinetobacter baumannii* Multidrug Efflux Pump Belonging to the MATE Family of Transporters. Antimicrob. Agents Chemother..

[32] Leus I.V., Weeks J.W., Bonifay V., Smith L., Richardson S., Zgurskaya H.I. (2018). Substrate Specificities and Efflux Efficiencies of RND Efflux Pumps of *Acinetobacter baumannii*. J. Bacteriol..

[33] Richmond G.E., Evans L.P., Anderson M.J., Wand M.E., Bonney L.C., Ivens A., Chua K.L., Webber M.A., Sutton J.M., Peterson M.L. (2016). The *Acinetobacter baumannii* Two-Component System AdeRS Regulates Genes Required for Multidrug Efflux, Biofilm Formation, and Virulence in a Strain-Specific Manner. Mbio.

[34] Yoon E.-J., Courvalin P., Grillot-Courvalin C. (2013). RND-Type Efflux Pumps in Multidrug-Resistant Clinical Isolates of *Acinetobacter baumannii*: Major Role for AdeABC Overexpression and AdeRS Mutations. Antimicrob. Agents Chemother..

[35] Yoon E.-J., Balloy V., Fiette L., Chignard M., Courvalin P., Grillot-Courvalin C. (2016). Contribution of the Ade Resistance-Nodulation-Cell Division-Type Efflux Pumps to Fitness and Pathogenesis of *Acinetobacter baumannii*. Mbio.

[36] Chen H., Cao J., Zhou C., Liu H., Zhang X., Zhou T. (2017). Biofilm Formation Restrained by Subinhibitory Concentrations of Tigecyclin in *Acinetobacter baumannii* Is Associated with Downregulation of Efflux Pumps. Chemotherapy.

[37] Alav I., Sutton J.M., Rahman K.M. (2018). Role of bacterial efflux pumps in biofilm formation. J. Antimicrob. Chemother..

[38] Hershberg R. (2017). Antibiotic-Independent Adaptive Effects of Antibiotic Resistance Mutations. Trends Genet..

[39] Jung H.-W., Kim K., Islam M.M., Lee J.C., Shin M. (2020). Role of ppGpp-regulated efflux genes in *Acinetobacter baumannii*. J. Antimicrob. Chemother..

[40] Lin M.-F., Lin Y.-Y., Yeh H.-W., Lan C.-Y. (2014). Role of the BaeSR two-component system in the regulation of *Acinetobacter baumannii* adeAB genes and its correlation with tigecycline susceptibility. BMC Microbiol..

[41] Henry R., Crane B., Powell D., Lucas D.D., Li Z., Aranda J., Harrison P., Nation R.L., Adler B., Harper M. (2015). The transcriptomic response of *Acinetobacter baumannii* to colistin and doripenem alone and in combination in an in vitro pharmacokinetics/pharmacodynamics model. J. Antimicrob. Chemother..

[42] Fernando D., Zhanel G., Kumar A. (2013). Antibiotic Resistance and Expression Of Resistance-Nodulation-Division Pump- and Outer Membrane Porin-Encoding Genes inAcinetobacterSpecies Isolated from Canadian Hospitals. Can. J. Infect. Dis. Med. Microbiol..

[43] Amer M., Wasfi R., Attia A., Ramadan M. (2021). Indole Derivatives Obtained from Egyptian *Enterobacter* sp. Soil Isolates Exhibit Antivirulence Activities against Uropathogenic *Proteus mirabilis*. Antibiotics.

[44] Yaikhan T., Chuerboon M., Tippayatham N., Atimuttikul N., Nuidate T., Yingkajorn M., Tun A.W., Buncherd H., Tansila N. (2019). Indole and Derivatives Modulate Biofilm Formation and Antibiotic Tolerance of Klebsiella pneumoniae. Indian J. Microbiol..

[45] Sanchez-Carbonel A., Mondragón B., López-Chegne N., Peña-Tuesta I., Huayan-Dávila G., Blitchtein D., Carrillo-Ng H., Silva-Caso W., Aguilar-Luis M.A., del Valle-Mendoza J. (2021). The effect of the efflux pump inhibitor Carbonyl Cyanide m-Chlorophenylhydrazone (CCCP) on the susceptibility to imipenem and cefepime in clinical strains of *Acinetobacter baumannii*. PLoS ONE.

[46] Ikonomidis A., Tsakris A., Kanellopoulou M., Maniatis A., Pournaras S. (2008). Effect of the proton motive force inhibitor carbonyl cyanide-*m*-chlorophenylhydrazone (CCCP) on *Pseudomonas aeruginosa* biofilm development. Lett. Appl. Microbiol..

[47] Zhang Y., Li Z., He X., Ding F., Wu W., Luo Y., Fan B., Cao H. (2018). Overproduction of efflux pumps caused reduced susceptibility to carbapenem under consecutive imipenem-selected stress in *Acinetobacter baumannii*. Infect. Drug Resist..

